# Identification of polymorphic inversions from genotypes

**DOI:** 10.1186/1471-2105-13-28

**Published:** 2012-02-09

**Authors:** Alejandro Cáceres, Suzanne S Sindi, Benjamin J Raphael, Mario Cáceres, Juan R González

**Affiliations:** 1 Center for Research in Environmental Epidemiology (CREAL), and Institut Municipal d'Investigació Mèdica (IMIM), Barcelona 08003, Spain; 2 CIBER Epidemiología y Salud Pública (CIBERESP), Barcelona 08003, Spain; 3 Department of Molecular Biology, Cellular Biology and Biochemistry, and Center for Computational Molecular Biology, Brown University, Providence, RI 02912, USA; 4 Department of Computer Science, and Center for Computational Molecular Biology, Brown University, Providence, RI 02912, USA; 5 Institut de Biotecnologia i de Biomedicina, Universitat Autonoma de Barcelona, 08193 Bellaterra, and Institució Catalana de Recerca i Estudis Avancats (ICREA), 08010 Barcelona, Spain

## Abstract

**Background:**

Polymorphic inversions are a source of genetic variability with a direct impact on recombination frequencies. Given the difficulty of their experimental study, computational methods have been developed to infer their existence in a large number of individuals using genome-wide data of nucleotide variation. Methods based on haplotype tagging of known inversions attempt to classify individuals as having a normal or inverted allele. Other methods that measure differences between linkage disequilibrium attempt to identify regions with inversions but unable to classify subjects accurately, an essential requirement for association studies.

**Results:**

We present a novel method to both identify polymorphic inversions from genome-wide genotype data and classify individuals as containing a normal or inverted allele. Our method, a generalization of a published method for haplotype data [1], utilizes linkage between groups of SNPs to partition a set of individuals into normal and inverted subpopulations. We employ a sliding window scan to identify regions likely to have an inversion, and accumulation of evidence from neighboring SNPs is used to accurately determine the inversion status of each subject. Further, our approach detects inversions directly from genotype data, thus increasing its usability to current genome-wide association studies (GWAS).

**Conclusions:**

We demonstrate the accuracy of our method to detect inversions and classify individuals on principled-simulated genotypes, produced by the evolution of an inversion event within a coalescent model [2]. We applied our method to real genotype data from HapMap Phase III to characterize the inversion status of two known inversions within the regions 17q21 and 8p23 across 1184 individuals. Finally, we scan the full genomes of the European Origin (CEU) and Yoruba (YRI) HapMap samples. We find population-based evidence for 9 out of 15 well-established autosomic inversions, and for 52 regions previously predicted by independent experimental methods in ten (9+1) individuals [3,4]. We provide efficient implementations of both genotype and haplotype methods as a unified R package inveRsion.

## Background

Inversions have long been known to play an important role in chromosomal evolution [5]. Indeed, large inversions are thought to contribute to speciation through reproductive isolation caused by reduced recombination between normal and inverted chromosomes [6]. Inversions of a wide range of sizes are abundant in mammalian lineages [7]. Additionally, extensive study of inversions in *Drosophila *revealed that inversions can leave genetic signatures, such as reduced nucleotide variation, within the inverted region [8-11]. More recently, many polymorphic inversions have also been found in humans [3,4,12]. A number of these have functional consequences; polymorphic inversions have been associated with genetic disorders [13], complex disorders such as asthma [14] and even positive selection [15].

Recent resequencing efforts of many human genomes continue to reveal the prevalence of structural variation in humans [16]. However, despite decreases in the cost of sequencing, genotyping microarrays remains the most cost-effective technology for analyzing entire genomes on thousands of individuals. Moreover, inversions have been traditionally difficult to study using experimental techniques. The typical presence of large inverted repeats at the breakpoints is a major challenge for their detection even with current next-generation sequencing techniques. While only few inversions (~ 15) have been experimentally validated [11,13,15,17-22], a few studies have scanned the whole genome providing experimental evidence for a number of candidate regions. For example, Levy and colleagues determined inverted regions by the whole genome assembly of one subject [3], and Kidd el al. used fosmid paired-end mapping in nine individuals [4]. As such, the ability to accurately predict new inversions and infer their status on large number of subjects would provide a valuable tool for clinical and evolutionary studies of human populations.

Previous studies have used haplotype tagging to indirectly infer which chromosomes are most likely to have an inversion, assuming recombination suppression in inverted heterozygous. For instance, Steffanson et al. [15] showed that an inversion within 17q21 in the European population can be tagged with two different haplotype groups (H1/H2), each related to a polymorphic variant of the *MAPT *gene. Although haplotype tagging is performed on large groups, it is suitable for regions known to have inversions and known to exhibit divergence between the two arrangements. Three studies [13,23,24], for instance, identified two haplotype groups within a region containing the known 17q21 and 8p23 inversions, and then experimentally validated the tagging on selected samples of the HapMap population. Taken together, this small group of subjects can be used to validate newly developed methods that determine the status of inversions in individuals.

Using a different approach, based on differences in linkage between groups of SNPs, two other methods have been developed to discover the presence of inversions across the genome [1,25]. Bansal et al. [25] used differences in linkage disequilibrium (LD) to determine regions likely to be inverted. However, their method requires the human reference to contain the minor allele, and does not predict which chromosomes in the population are most likely to have the inversion; a factor that is essential for association studies. More recently, Sindi and Raphael [1] developed a probabilistic method that models the population as a mixture of normal and inverted haplotypes, and thus had increased power to detect inversions of lower frequency. Although their method accurately predicts inversion frequencies, it did not yield an accurate classification of individuals into normal and inverted subpopulations. The computationally intensive search of both methods have, in particular, failed to identify inversions like the one within 17q21 in the CEU population, for which a clear extended LD has been shown [13]. In addition, both methods were developed for haplotype (phased) data only.

One way to analyze genotype data, using this method, is to phase the entire genome and then apply it to the resulting haplotypes. This procedure is computationally demanding. For instance, it has been reported that compiled software like fastPHASE [26] can take up to 9 h to analyze 60 subjects in a 41,018 SNPs chromosome (3-GHz Xeon processor with 1 GB). Therefore, a method that directly analyzes genotypes, incorporating the limited phasing required by inversion detection, can substantially reduce this computational load and allow the complete implementation of the methodology in a single software tool to be used in standard up-to-date machines.

In this work, we propose a new methodology to (1) efficiently detect inversions across the genome by directly using genotype data and (2) accurately classify individuals in the population according to inversion status. In addition, our generalization of the inversion model for haplotypes [1], as a computational technique, allows us to treat the different problem of phasing haplotype blocks separated at any distance. Our new application of the inversion model, within the analysis of polymorphic inversion from genotypes, increases the applicability of the method to current GWAS, and enhances its usability by a higher computational efficiency. We provide an efficient implementation of the novel analysis of genotypes, and the new classification and search methods in the R package inveRsion, freely available through Bioconductor [27] (http://www.bioconductor.org/packages/devel/bioc/html/inveRsion.html). We also include a computationally improved version of the previous haplotype model, and the use of the Bayesian Information Criterion (BIC) to gather statistical evidence from neighboring regions.

Both prior LD studies [1,25] tested their methods by constructing "artificial" inversions by reversing the order of SNPs in phased haplotypes from HapMap. We provide a more rigorous test of our method by employing a recent software tool, invertFREGENE [2], that utilizes coalescent theory and suppression of recombination between inverted and normal chromosomes to produce artificial haplotype and genotype data. Lastly, we apply our method to HapMap Phase III data, where we compare the analysis of genotypes with that for haplotypes, assess the our classification accuracy in two validated inversions and search the whole genome for inversion signals.

## Methods

### The Inversion Model

The inversion model was first proposed by Sindi et al. 2010 [1] to predict inversion polymorphisms from phased data. Here we give a general formulation that allows us, in addition, to solve the distinct problem of phasing genotype data across inversion breakpoints, and thus reduce the computation load of phasing the whole genome.

We are given *m *independent observations of four random variables *B*_1_, *B*_2_, *B*_3_, and *B*_4_, ordered according to location in the reference genome. We are interested in assessing the likelihood of an inversion in the population with one breakpoint between markers *B*_1 _and *B*_2 _and the other breakpoint between markers *B*_3 _and *B*_4_. In the case an inversion exists, we expect to observe more than one subject *i *for which the formation of the two pairs (*B*_1_(*x_i_*), *B***_2_**(*x_i_*)) and (*B***_3_**(*x_i_*), *B*_4_(*x_i_*)) is less probable than that of (*B*_1_(*x_i_*), *B***_3_**(*x_i_*)) and (*B***_2_**(*x_i_*), *B*_4_(*x_i_*)), within the population sample (*i *= 1...*m*). We denote the joint random variables as *B*_1,2_(*x_i_*), *B*_3,4_(*x_i_*), *B*_1,3_(*x_i_*) and *B*_2,4_(*x_i_*) respectively.

In a sample where no individual is inverted, we assume that the probability *P_fwd_*(*x_i_*|*n*_1,2_, *n*_3,4_) of the observed values *B*_1_, *B*_2_, *B*_3 _and *B*_4 _at *x_i _*can be factored as the product between the probability of the pairs (*B*_1_(*x_i_*), *B*_2_(*x_i_*)) and (*B*_3_(*x_i_*), *B*_4_(*x_i_*))

(1) $$P_{n_{1,2}}\left(B_{1,2}\left(x_{i}\right)\right)\times P_{n_{3,4}}\left(B_{3,4}\left(x_{i}\right)\right).$$

where *n*_1,2 _and *n*_3,4 _indicate the respective probabilities of observations for *B*_1,2 _and *B*_3,4_. The likelihood of all the *m *independent observations is then

(2) $$L_{0}\left(x\text{|}n_{\text{1,2}},n_{\text{3,4}}\right)=\overset{m}{\underset{k=1}{\prod }}P_{fwd}\left(x_{i}\text{|}n_{1,2},n_{3,4}\right).$$

On the other hand, in a population where all observations are inverted, the probability *P_inv_*(*x_i_*|*r*_1,3_, *r*_2,4_) of obtaining the specific values of the *x_i _*observation is

(3) $$P_{r_{1,3}}\left(B_{1,3}\left(x_{i}\right)\right)\times P_{r_{2,4}}\left(B_{2,4}\left(x_{i}\right)\right),$$

where, as before, *r*_1,3 _and *r*_3,4 _correspond to the probabilities of observations for *B*_1,3 _and *B*_2,4_.

In a real population sample, where we expect a mixture of forward and inverted individuals with mixture frequency *π*. In addition, given appropriate probabilities for all four joint random variables, *f*_1,2_, *f*_3,4 _for the forward subpopulation and *r*_1,3_, *r*_2,4 _for the inverted subpopulation, and *ω *= (*f*_1,2_, *f*_3,4_, *r*_1,3_, *r*_2,4_), the probability of observing the values of *B*_1_, *B*_2_, *B*_3 _and *B*_4 _for an arbitrary *x_i _*is

(4) $$\begin{array}{ccc} P_{mixture}\left(x_{i}\text{|}\omega \right) & =\left(1-\pi \right)P_{fwd}\left(x_{i}|f_{1,2},f_{3,4}\right) & \\ & +\pi P_{inv}\left(x_{i}|r_{1,3},r_{2,4}\right). \end{array}$$

with population sample likelihood

(5) $$L_{1}\left(x\text{|}\omega \text{,}\pi \right)=\overset{m}{\underset{k=1}{\prod }}P_{mixture}\left(x_{i}\text{|}\omega ,\pi \right).$$

An important quantity in predicting the inversion status of an individual is the relative probability that the individual belongs to the forward subpopulation:

(6) $$r_{0,i}=\frac{P_{fwd}\left(x_{i}|f_{1,2},f_{3,4}\right)}{P_{fwd}\left(x_{i}|f_{1,2},f_{3,4}\right)+P_{inv}\left(x_{i}|r_{1,3},r_{2,4}\right)},$$

this is also called the responsibility to the forward model. Similarly, the responsibility of an individual to the inversion model, *r*_1,*i *_= 1 - *r*_0,*i*_.

We test the hypothesis that *π *= 0 (null model) vs. *π >*0 (alternative model) with a maximum likelihood approach. We maximize the likelihood of the data under each model; that is, for the null model we use the empirical distribution of allele frequencies for the null model and for the alternative model we select the allele frequencies and inversion frequency *π *using the Expectation-Maximization (EM) algorithm for mixtures of probability distributions. The decision to reject the null model (no inversion) for the alternative model is based on a model selection test. In this work, we utilize the Bayesian Information Criterion (BIC) to both identify candidate inversions and classify individuals carrying the polymorphic inversions.

### Inversion model for genotype data

In the analysis of genotype data, we apply the inversion model in two steps. In the first step, we use the inversion model to phase and pair haplotype blocks around potential breakpoints. This approach allows flexibility to phase two haplotype blocks at any distance, without phasing the whole genome or constantly re-phasing regions, when one of the breakpoints is changed. This use of block phasing is a novel application of the inversion model. The second step is the original application of the model [1] to identify which individuals are likely to have a genetic inversion.

In this work, we define a candidate breakpoint as a pair of consecutive SNPs. A segment tested for an inversion is thus defined by two candidate breakpoints (left and right). We flank the left and right candidate breakpoints by two blocks of *N *SNPs each, and perform local haplotype phasing with haplo.stats [28]. As a result, an individual *i *has two haplotypes, *L*_1_(*x_i_*) and *L*_2_(*x_i_*) of 2*N *SNPs each, *containing *the left breakpoint in the middle, and two 2*N *SNPs haplotypes *R*_1_(*x_i_*) and *R*_2_(*x_i_*), *containing *the right breakpoint in the middle. The first application of the inversion model is to sort out the chromosome pairing of the containing blocks, that is, we set *B*_1 _= *L*_1_, *B*_2 _= *R*_1_, *B*_3 _= *R*_2_, *B*_4 _= *L*_2 _and *m *= *n_s _*(number of subjects) in the inversion model. The responsibilities for the alternative model tell us if the pairing of (*L*_1_, *R*_1_) in one chromosome and (*R*_2_, *L*_2_) [e.g. (*L*_2_, *R*_2_) ordered by handedness] in the other chromosome is more (less) likely than the pairing (*L*_1_, *R*_2_) and (*R*_1_, *L*_2_) [(*L*_2_, *R*_1_)], for each subject in the sample. This first process phases the genotype data between any pair of blocks, surrounding candidate breakpoints.

The second application of the inversion model is to determine the presence of genetic inversions on phased data, as originally proposed in Sindi and Raphael 2010 [1]. In total, we have *n_c _*= (2 * *n_s_*) haplotype blocks phased by the former pairing. We first aggregate the data into two variables one for each breakpoint, *H_L_*(*L_j_*(*x_i_*)) corresponds to the haplotype of subject *i *and chromosome *j *containing the left breakpoint, and *H_R_*(*R_j_*(*x_i_*)) to the haplotype of subject *i *and chromosome *j *containing the right breakpoint. We next split the left haplotype into two *N *- SNPs blocks (*H_LL_*, *H_LR_*) = *H_L _*each of which flank the potential left breakpoint, and (*H_RL_*, *H_RR_*) = *H_R _*each of which flank the right breakpoint. In the inversion model, we set *B*_1 _= *H_LL_*, *B*_2 _= *H_LR_*, *B*_3 _= *H_RL_*, *B*_4 _= *H_RR _*and *m *= *n_c_*, as the total number of chromosomes in the sample. The responsibilities of this model now determine for which chromosomes the pairings (*H_LL_*, *H_LR_*) and (*H_RL_*, *H_RR_*) are more (less) likely than the pairings (*H_LL_*, *H_RL_*) and (*H_LR_*, *H_RR_*). That is, the model determines which chromosomes are likely to be inverted between the left and right breakpoints considered.

An important consideration in our analysis is whether the initial phasing step itself is confounded by the presence of an inversion. Thus, in the first step of our analysis, we perform two other (more complex) local haplotyping strategies. In the first strategy, we locally haplotype each *N*-SNP flanking block separately with haplo.stats and then use the inversion model to phase the internal flanking blocks, i.e. those assumed to be within the inversion segment. Finally we use the model again to phase the internal haplotype with each external block. A second alternative strategy is to perform the local haplotyping with haplo.stats once on the surrounding blocks following the forward population and twice following the inverted population, leaving two distinct haplotype data for each population. We compare the accuracy of each phasing strategy in the prediction of inversions on simulated data and contrast them with the accuracy of the prediction for already phased data.

### Inversion Detection and Subject Classification

In addition to providing a novel method to analyze genotype data for polymorphic inversions directly, our work aims at improving the efficiency of detecting inversions, and the accuracy of classifying individuals with respect to inversion status.

Previous LD methods [1,25] considered a potential breakpoint between every pair of adjacent SNPs in the genome with sufficient diversity and physical distance between breakpoints. Thus, the total number of segments tested for inversions was very large, *O*(*n*!) where *n *corresponds to the number of candidate breakpoints or SNPs. To reduce the computational load, we decrease the number of regions tested by using a sliding window. We tested our approach on simulated inversions from invertFREGENE [2], and found our model still correctly identifies an inversion if the candidate segment tested is contained within the real inversion sequence and "large enough" compared to the real inversion. Consequently, we scan the chromosome with trial segments of fixed length (window size), as probes for detecting inversions with comparable length. We then reconstruct the true inversion by considering all trial segments with sufficiently large Bayesian Information Criterion (BIC). This simplification massively reduces the number of computations to *O*(*n*).

In addition, prior methods for haplotype data were unable to successfully identify individuals carrying the inversion, a necessity for genome association studies. We develop an accurate classification method based on the responsibility of each individual to the normal or inverted subpopulation in overlapping windows. For a fixed window *w *we determine the responsibility of chromosome *i*, to the forward model $r_{0,i}^{w}$. Sindi and Raphael [1] attempted to classify individuals based on their dominant responsibility, normal if $r_{0,i}^{w}\geq 0.5$ and inverted otherwise; however, this did not yield a useful classifier because the classification from any single instance of the mixture model was poor. We find that combining classifications from adjacent, overlapping windows yields a substantial improvement in classification accuracy with the true inversion. More generally, we estimate the responsibility of individual *i *to the inversion *I *by a majority vote overall overlapping segments where *BIC *exceeds a given threshold *BIC > t_B_*:

(7) $$r_{0,i}^{I}\left(t_{B}\right)=\frac{\sum_{w}\text{H}\left(r_{0,i}^{w}-0.5\right)\text{H(}BIC_{w}-t_{B}\text{)}}{\sum_{w}\text{H(}BIC_{w}-t_{B}\text{)}},$$

where H is the step Heaviside function, *H*(*z*) = 1 when *z >*0 and 0 otherwise. For haplotype data, we classify a chromosome *i *as inverted when $r_{0,k}^{I}\left(t_{B}\right)<0.5$ for a particular value of *t_B_*. For genotype data, we classify each individual as homozygous for the reference orientation, heterozygous for the inversion or homozygous for the inversion, depending on the classification obtained from the responsibilities of both chromosomes. We define the *classification accuracy *of our method as the number of individuals with correctly identified inversion status (i.e., fraction of true positives and true negatives). In the simulation results below, we compare the classification accuracy of our method to the true known categorization of individuals as function of *t_B_*. In practice, our classification accuracy increases with *t_B_*.

### Datasets

We studied the power of our method, implemented as the R package inveRsion, to detect inversions on both haplotype and genotype data by analyzing previously known and simulated polymorphic inversions.

First, we applied our method to simulated haplotypes and genotypes to asses our performance at both detecting inversions and classifying individuals into normal and inverted subpopulations. Using recently developed software, invertFREGENE [2], we simulated inversions of varying lengths (0.1 - 1.0 Mb) and varying frequencies *π *= 10 - 90% in a populations of 1,000 individuals (2,000 chromosomes). The software provided haplotypes, genotypes and inversion status of each chromosome in the population. Parameters used in running the software, such as number of generations to achieve equilibrium, and rates of recombination and mutation, were taken without change from [2]. In particular, the segments simulated by invertFREGNE have a median fraction of heterozygous of 0.34 and overall SNP density of 0.33 Kbase/SNP on a 2 Mb segment.

To check the performance of our method on real data, we used the SNP data from HapMap phase III (http://www.hapmap.org), totalling 1.5 M SNPs on 1184 individuals from 11 different populations. As a first step, we analyzed both haplotype and CEU and YRI populations from chromosomes 16 and 17, where well characterized polymorphic inversions have been found. Next, we performed a region of interest analysis, where we did a detailed search around two previously identified inversions (17q21 and 8p23), and classified subjects for all 11 HapMap III populations by predicted inversion status. We then validated the inversion genotypes on previously reported individuals. Finally, for the more demanding task of scanning the whole genome, we used the SNP genotype data of the joint CEU+YRI population (201 subjects). Significant regions were compared with a set of previously reported inversions. We used a first set of 15 well characterized and experimentally validated inversions, and then a second set of putative inversions predicted by whole genome assembly comparison [3] or paired-end mapping experiments [4].

### Implementation

Some technical considerations were made in the implementation of the previously described models in the inveRsion R package. In analyzing genotypes, we removed SNPs with missing values for more than 10% of the individuals. For the remaining SNPs, missing values were imputed during local phasing with haplo.stats, we selected haplotypes with the highest posterior probability at each point, and did not considered the propagation of this error into the inversion model. To further limit the regions tested, we only considered breakpoints between SNPs where at least one had minor allele frequency ≥ 10%.

For both mixture models, we used the empirical distribution of allele frequencies as the initial condition for the EM algorithm. We conjectured that this choice would be appropriate to locate the global minimum, and verified this conjecture on simulated inversions by running the EM algorithm with randomly chosen initial conditions.

## Results

### Simulated data

#### Characterization of a single inversion

**Figure 1:**
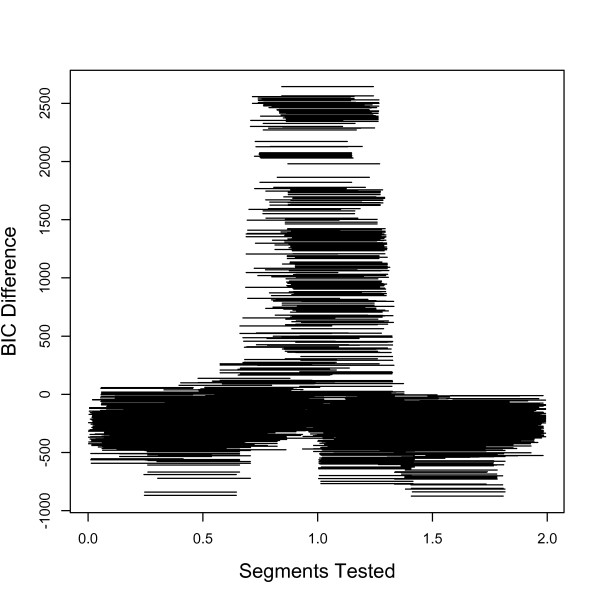
**Scan search for inversions in simulated data**. We show the Bayesian information criterion (BIC) for each possible window (fixed size 0.4 Mb) for genotype data on a simulated inversion with 40% frequency located at position 0.75-1.25 Mb. Our method clearly favors rejecting the null (no inversion) model (when BIC*>*0) in the region of inversion.

We first ran our inversion model for the genotype data produced by invertFREGENE [2] on one inversion. Using a fixed window size of 0.4 Mb, and 5 SNPs flanking each potential breakpoint, inveRsion successfully detected the true inversion between 0.75 - 1.25 Mb, see Figure 1. As expected, many segments overlapping the true inversion favor the alternative (inversion) model over null model.

Because we know which chromosomes carry the inversion, we directly assess our classification accuracy (i.e., fraction of individuals with correctly identified inversion status). We show the median classification accuracy as function of *t_B _*(equation 7) for a run of 30 invertFREGENE simulations. Note that classifications for haplotype data are at the chromosome level and for genotype data are at the subject level. For some cases, using low values of *t_B _*our method identified extra or spurious regions of interest; however, in general as *t_B _*increased, the classification improved.

**Figure 2:**
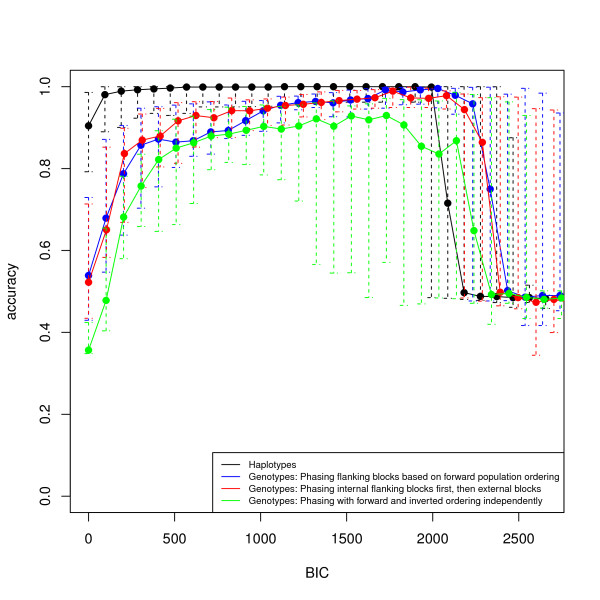
**Accuracy in classification of chromosomes (haplotype data) and subjects (genotype data) into normal and inverted subpopulations**. We compute the classification of individuals according to Equation 7 using the sliding window segments, from Figure 1 that overlap and have BIC *> t_B _*in one simulation sample. We compare results for the our method on haplotypes and three local phasing procedures for genotype data. Error bars indicate data within the second and third quartile.

In Figure 2, we show the median classification accuracy for windows overlapping the inversion simulated in Figure 1. For each candidate window, we determined the responsibility of each individual, $r_{0,k}^{w}$, and computed the classification of individuals for increasing values of the BIC threshold *t_B_*. For both haplotype and genotype data, our ability to successfully classify individuals improves with increasing threshold *t_B_*. Reduced accuracy for genotype data, compared to haplotype data, is expected because of errors accumulating from local phasing and incorrect pairing of local haplotypes. In this example, for haplotype data, high accuracy is quickly obtained with perfect classification for *BIC *~ 500. For genotype data we show the results of three local phasing strategies. We see that phasings based on the forward population and internal flanking blocks have comparable accuracies which are optimized at *BIC *~ 1700. The strategy of haplotyping the forward and inverted populations separately can be highly accurate (0.9) but is consistently lower than the other two. This can be due to the increased amount of computation this strategy requires. Overall, we found no accuracy improvement over our initial simplified phasing strategy.

Decreased accuracies for both genotype and haplotype data for the highest *t_B _*values may be attributed to fewer observations. Indeed, the decay of accuracy at highest *t_B _*depicts the classification difficulties from prior methods [1] that used only the single most likely predicted region to classify individuals. Overall, our weighted method of classification based on BIC values is more robust than selecting a classification based on a single segment with likelihood ratio of lowest p-value.

#### Classification and detection performance

**Figure 3:**
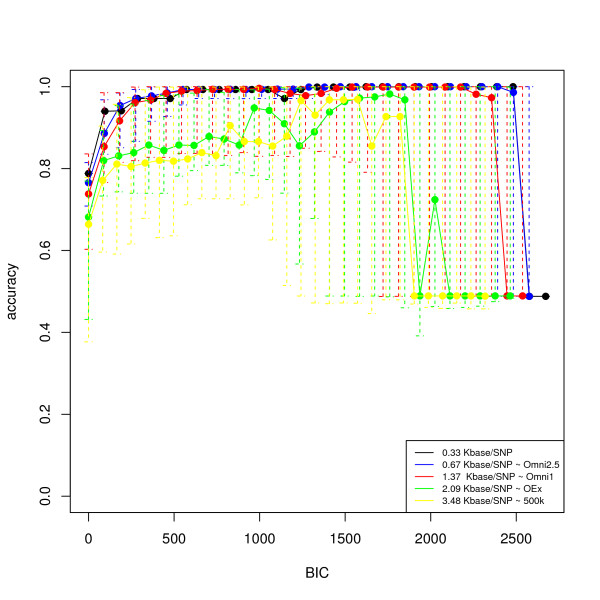
**Average accuracy in the classification of subjects (haplotypes) into normal and inverted populations**. The picture shows the average and individual classification accuracies across simulated cases (50) as a function of *t_B_*, for different SNP densities.

We first analyzed the ability of our model to determine simulated inversions on haplotype data with five different SNP densities, matching some of those in current Illumina chip technologies. Figure 3 shows the classification accuracy of inversion status for the inversions specified in the previous sections. We can see that our method performs well at all the densities studied, with more accuracy at higher densities. At the level of the 500 k arrays we can still achieve high chromosome classification accuracy.

**Figure 4:**
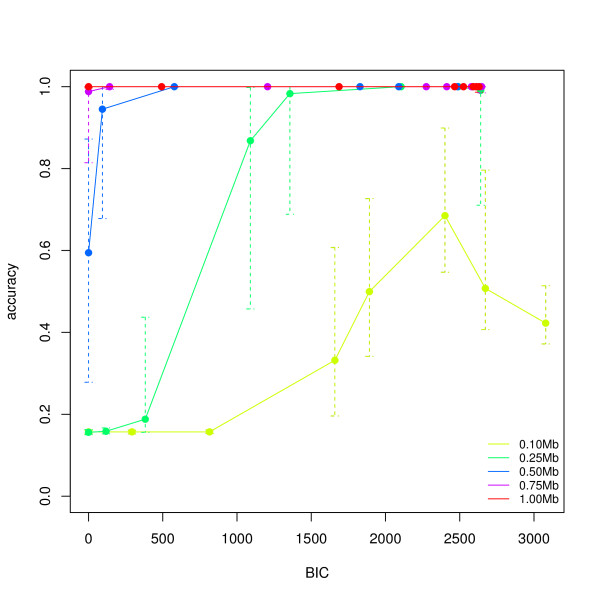
**Average accuracy in the classification of subjects (genotypes) into normal and inverted populations**. The picture shows the average and individual classification accuracies across simulated cases (50) as a function of *t_B_*, for different lengths and population frequency of 60%. The simulated regions were scanned with a window size of 60% of the real inversion and block size of 5 SNPs.

To assess the performance of our method to detect inversions and classify individuals in genotype data, we simulated samples varying the length of the inversion and its population frequency. We ran invertFREGENE 50 times for each condition, and computed classification accuracy for different *t_B _*values (Figure 4), segmental sensitivity of detection (percentage of the true segment identified -Additional file 1, Figure S1) and segmental false discovery rate (percentage of the segment identified that is not part of the true segment -Additional file 1, Figure S2). We used a window size of 60% of the inversion length and flanking blocks of 5 SNPs (*N *= 5). A total a 50(*cases*) × 5(*lengths*) × 9(*frequencies*) = 2250 simulations were run. Figure 4 shows the average accuracy of each subject's inversion genotype classification for 5 different lengths, and a population frequency of 60%. At this frequency, high accuracies *>*0.90 are achieved for lengths greater than 0.25 Mb when *t_B _*is large enough. In particular, our method is most accurate for larger inversions.

**Figure 5:**
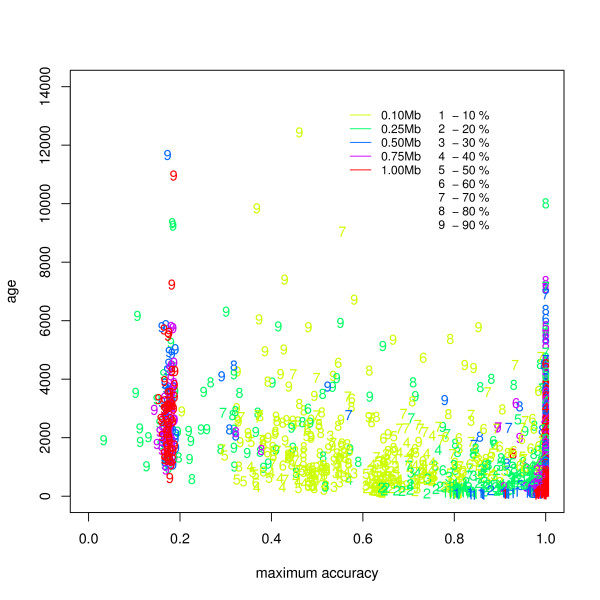
**Maximum accuracy in the classification of subjects (genotypes) into normal and inverted populations as function of inversion age**. The figure shows mean classification accuracy across *t_B _*values for all the simulation cases (2250). Our method achieves high accuracy for large inversions and a wide range of frequencies, as simulated by invertFREGENE. We find low accuracy in a discontinuous cluster of older and high frequency inversions, suggesting a critical behavior in the simulations at this point which we discuss further in the main text.

We also studied the classification accuracy at detecting inversions of all frequencies in relation to the age of the inversion, as treated in invertFREGENE. Figure 5 shows the age of the inversion as function of maximum accuracy across *t_B _*for each simulated case. We see that our method achieves highest accuracy for larger lengths and at a wide range of frequencies. Our method is less accurate at identifying older inversions with frequencies (*>*80%). Particularly, we observe that inversions at 90% frequency heavily cluster in a low predictive range, indicating a critical phenomena in the simulations for frequency increments at high values.

Additional file 1, Figures S1 and S2, illustrates the segmental sensitivity and false discovery rate (FDR) for these simulations, showing the ability of the method to accurately identify the region containing the inversion. We see that while we have high sensitivity in a large range of BIC thresholds (*>*0), the FDR decreases with increasing *t_B_*. Therefore, all the inversion lengths studied are detectable, and higher accuracy is achieved for inversions *>*0.5*Mb*. To assess false inversion detection, we ran simulations (250 cases) with no inversions and scanned them with three different window sizes (0.2, 0.4 and 0.6 Mb). In each case we identified the top 2% *t_B _*quantiles for which at least one region of interest (overlapping windows) was found. Histograms of for all three scans are shown in Additional file 1, Figure S3. In particular, we find that, in samples of 1000 subjects, an FDR of 0.05 is achieved at *t_B _*= 286 for scans of 0.6*Mb*, *t_B _*= 718 for 0.4*Mb *and *t_b _*= 2400 for 0.2*Mb*.

### HapMap data

#### Genotype and haplotype (phased) data

We analyzed the CEU and YRI populations from HapMap Phase III. We initially performed a scan on chromosome 16 for the CEU, and chromosome 17 for the CEU and YRI populations, to compare the results obtained for genotype and haplotype (phased) data. We used two types of genotype data; the original genotypes before phasing and reconstructed genotypes from the phased haplotypes, with the encoding of 0, 1 and 2. We examined chromosomes 16 and 17 with two window sizes (0.4 Mb and 0.7 Mb), and used a BIC threshold of *t_B _*= 50. We computed the inversion frequency of the estimated inversion from the majority vote for each chromosome in the sample.

Table 1 shows the results for the phased haplotype and genotype data for chromosome 16 of the CEU population. For the haplotype case, we find evidence on a experimentally validated inversion ~ 28.2 - 28.8 Mb [22], which is lost in the analysis of the genotype data. However, notice that on each dataset our method predicts an inversion with breakpoints at roughly ~ 34 - 35 Mb that closely match an inversion reported by paired-end mapping in the CEU population at 16p11.2 - 16p11.1 [4]. We achieved consistent results when analyzing the genotypes that were reconstructed from the reported haplotypes (Additional file 1, Table S1). This result endorses further experimental characterization of the region.

**Table 1:** Inverted sequences found in chromosome 16 of the CEU population using haplotype (phased) and genotype data

Data type	window	LBPmin	LBPmax	RBPmin	RBPmax	MaxBic	invFreq	Ns
Haplotypes	0.4	28.23496	28.40377	28.67367	28.80467	117.29	0.60	118
	0.4	33.70440	34.67086	34.11169	35.07705	153.01	0.39	494
	0.4	45.68583	46.08754	46.08754	46.49553	236.34	0.77	1302
	0.4	66.24902	66.44043	66.65147	66.84146	189.82	0.40	350
	0.4	68.51016	68.66370	68.91604	69.06441	159.35	0.42	641
	0.4	70.99660	71.05418	71.39778	71.45419	99.61	0.40	17
	0.7	33.49144	34.31572	34.19297	35.03194	207.00	0.49	923
	0.7	45.69871	45.81018	46.40781	46.51082	186.17	0.74	259

Genptypes	0.4	34.07920	34.55067	34.48884	35.00029	137.04	0.19	6
	0.4	68.51016	68.66370	68.93968	69.06441	147.57	0.46	38

The segments where identified scanning the whole chromosome with window sizes 0.4 Mb and 0.7 Mb. Overlapping trial segments with *BIC > *50 where selected for the final classification of chromosomes and to set the limits of the inverted sequences. Keys: LBPmin: minimum left breakpoint coordinate, LBPmax: maximum left breakpoint, RBPmin: minimum right breakpoint, RBPmax: maximum right breakpoint, MaxBic: maximum BIC, invFreq: frequency of the inversion within the population, Ns: Number of overlapping segments in the inversion.

We analyzed chromosome 17 for both the CEU and YRI populations (Table 2) separately. We predict 3 inversions in the YRI population and 4 in the CEU population. Interestingly, the 3 regions in the YRI population are very similar to 3 of the 4 predictions in the CEU population, but with fewer overlapping sliding windows and different predicted inversion frequencies. Importantly, the remaining inversion predicted in the CEU population corresponds to a known inversion at 17q21, ≈ 40.4 - 42.4 Mb with inversion frequency of 20-25% in mixed European populations, and absent from the YRI HapMap sample [15]. To ensure the specificity of this predicted inversion to the CEU population, we reanalyzed the region 40 - 43 Mb in greater detail by considering all possible segments with length ≥ 0.7*Mb*. As expected, we did not find an inversion in the YRI population, but did obtain improved information on the inversion location in the CEU population: Left Breakpoint = (41.07267 Mb,41.47629 Mb), Right Breakpoint = (42.09285 Mb,42.17783 Mb), Maximum BIC = 167.91, Inversion Frequency = 0.22, Number of Overlapping Windows = 1638. Note that this inversion was not identified by prior studies using nucleotide variation data [1,25].

**Table 2:** Predicted inversions on chromosome 17 for the CEU and YRI populations on genotype data (window size 0.4*Mb *and *t_B _*= 50).

Population	LBPmin	LBPmax	RBPmin	RBPmax	MaxBic	invFreq	Ns
CEU	21.96788	22.09834	22.37082	22.50184	99.32	0.25	24
	24.96601	25.14566	25.36840	25.54785	137.18	0.47	265
	41.07267	41.64131	41.47280	42.09285	196.52	0.22	444
	53.84780	54.18923	54.25283	54.59168	322.95	0.28	802

YRI	21.88505	22.11686	22.36658	22.52599	111.94	0.39	9
	24.99465	25.09088	25.39824	25.49422	79.01	0.57	11
	53.90580	54.11198	54.30626	54.51477	235.38	0.46	12

One of the CEU predictions corresponds to a known inversion at 17q21, and was also found with window size 0.7*Mb *(see main text). Consistent with experimental evidence this is a CEU specific inversion, no inversions where predicted in the YRI population in this region with any window size.

#### Subject classification for inversions within 17q21 and 8p23

To accurately classify individuals according to inversion status for two known inversions (17q21 and 8p23) we preformed extensive searches near reported breakpoints. In both cases, we obtain high accuracy at classifying individuals with known inversion status.

First, we performed an extensive search on the region between 39-43 Mb in the chromosome 17 for the complete 11 HapMap III populations, with *t_B _*= 0. The region identified as the potential inversion had Left Breakpoint = (40.81726 Mb, 41.18244 Mb), Right Breakpoint = (42.09285 Mb, 42.1871 Mb), Maximum BIC = 1201.108, Inversion Frequency = 0.08 and was identified by 155 overlapping windows in the scan. Additional file 1, Figure S4, shows that windows favoring the inversion model (*BIC >*0) flank gaps in the SNP distribution. These gaps correspond to the segmental duplications which have been suggested to give rise to the inversion sequence that sits between them. We computed the classification of each chromosome in the sample with a *BIC >*600 that was the minimum BIC for which the classification was optimal for the subjects with known inversion status [13]. We correctly classified all 24 samples with known inversion status (7-CEU, 8-YRI, 4-CHB, 4-JPT) (Additional file 1, Table S2). That is, CEU subjects NA10847, NA12156, NA12813 were reported as heterozygous for the inversion and all other subjects as non-inverted homozygous. The inversion status of subjects from all 11 populations is shown in Table 3. We see that the chromosomal incidence of the inversion for the CEU in the joint population analysis is 26% consistent with the single population analysis. Finally, the cross tabulation in Table 4 between the inversion and the "rs1800547" SNP genotypes further validates the tagging of our inversion inference with this SNP (accuracy *>*0.99), as expected from their known relation to the H1/H2 haplotypes [15].

**Table 3:** Frequency of the inversion in 17q21 across all HapMap III populations

genInv	ASW	CEU	CHB	CHD	GIH	JPT	LWK	MEX	MKK	TSI	YRI
Hom	81	59	100	100	82	99	100	73	89	44	100
invHet	18	41	0	0	17	1	0	23	9	41	0
invHom	1	1	0	0	1	0	0	4	2	15	0

chr freq	0.10	0.26	0	0	0.10	0.01	0	0.17	0.06	0.44	0

Hom: non-inverted homozygous, Het: inverted heterozygous, and invHom: inverted homozygous. The chromosomal frequency of the inversion is highest in the European populations (CEU + TSI), see last row of the table. *Population Key*: ASW: African ancestry in Southwest USA, CEU: Utah residents with Northern and Western European ancestry, CHB: Han Chinese in Beijing, CHD: Chinese in Metropolitan Denver, GIH: Gujarati Indians in Houston, JPT: Japanese in Tokyo, LWK: Luhya in Webuye, MXL: Mexican ancestry in Los Angeles, MKK: Maasai in Kinyawa, TSI: Toscani, YRI: Yoruba in Ibadan.

**Table 4:** Tagging of the inversion in 17q21 as detected by inveRsion and SNP "rs1800547"

	no-inversion homozygous	inversion heterozygous	inversion homozygous
rs1800547 homozygous	985	0	0
rs1800547 heterozygous	9	166	0
rs1800547 variant-homozygous	0	1	23

The H1 and H2 haplotypes have been traditionally associated to the inversion status in the 17q21 inversion. From those haplotypes the SNP "rs1800547" from the *MAPT *gene has been used to tag the inversion in 17q21 [15]. As expected, our classification of individuals from all 11 HapMap populations agrees well with the their status for this SNP.

We next studied the more challenging classification of the recurrent inversion at 8p23 [13], for which no significant signal was obtained from the whole genome scan on the CEU+YRI sample. We performed an detailed search between 6-13 Mb of chromosome 8, and detected a signal (Maximum BIC = 345.349) within the inversion sequence. The region identified was between Left Breakpoint=(8.804291 Mb, 8.98084 Mb) and Right Breakpoint=(10.80516 Mb, 10.98203 Mb), see Additional file 1, Figure S5. We compared our classification with the true inversion status reported for 41 of the individuals from 4 of the 11 HapMap populations. (In Additional file 1, Table S3). We found that while the exact match of the subject genotypes is (70%), the subject-wise identification of an inversion presence is 88%. Population-wise, we see that the CEU are the best predicted with 91% accuracy, while the YRI sample has only 36% accuracy. In the experimental studies all 11 YRI subjects have an inversion, 7 of which are inversion heterozygous. None of the 4 JPT subjects in the experimental validation group have an inversion, and inveRsion classify them accordingly. However, we do not correctly identify any of the 6 CHB subjects carrying the inversion.

#### Genome-wide scan

**Figure 6:**
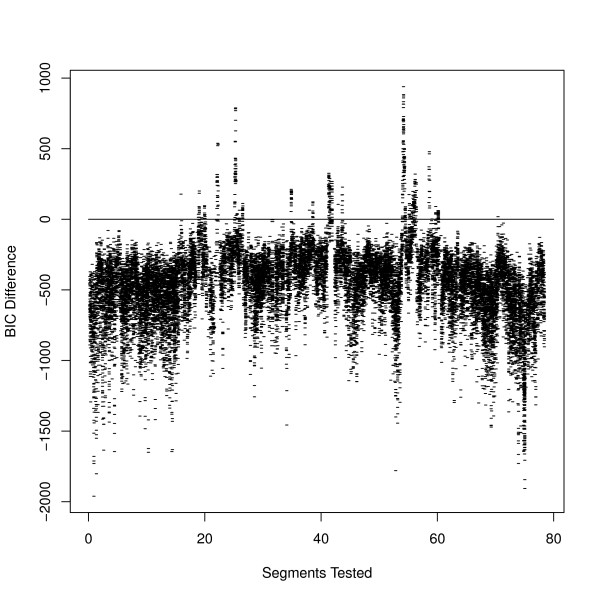
**Scan across chromosome 17 for the joint CEU + YRI populations**. We conduct an extensive local search for a known inversion on chromosome 17. We show the BIC values for each sliding window of size 0.4 Mb; a horizontal line indicates the zero level for which the inversion model favors the mixture of inverted and non-inverted populations. The inversion at 17q21 (~ 40 Mb) is clearly visible. The full list of inversions are given in Additional file 1, Table S4.

Lastly, we scanned the whole genome of the combined CEU and YRI populations of HapMap III with three window sizes (1 Mb, 0.7 Mb and 0.4 Mb) (Figure 6). Across all chromosomes, we found 263 candidate regions for inversions from overlapping windows with *t_B _*= 0. In Additional file 1, Table S4, we report the outer limits of the left and right break points of the inversions found. A number of inversions detected with window size of 1 Mb are also detected with lower sizes.

Although the median size of inversion reported by Kidd and colleagues 2008 is 0.1 MB, 23% of those segments are at least above 0.4 Mb, and thus are potentially detectable with our method. We find a total of 52 inversions (20% of our reported regions) that overlap either those found by Kidd et al. [4] on nine subjects or by Levy and colleagues [3] on one subject, providing additional evidence of their existence. A higher proportion, 89 inversions (33%) are at least 1 MB distance from a reported inversion.

Table 5 lists the 15 autosomal inversions that have been experimentally validated, 8 of which are detected in our genome-wide scan on genotype data. As mentioned above, the inversion on chromosome 16 reported by Martin et al. 2004 [22] was found on the analysis of haplotype data only. We note these inversions were previously identified in specific individuals, and our current studies now provide population-based evidence to these inversions. In particular, we fully recovered the inversions in the 17q21 and 15q24 regions and found candidate regions within 3 Mb distance from those within the 3q29, 5q13.3, 17q21.3 regions. We found independent evidence for each break point of the chromosome 10 inversion, and for the right break point of the inversion in 15q11. As we mentioned in the previous section, the evidence on 8p23 results from an extensive search on the region and not from a genome-wide scan.

**Table 5:** Experimentally validated inversions

CHR	Inv. Size (Mb)	Cyt.band	Source	Segment	inveRsion scan	Note
ch3	1.9	3q29	Antonacci 2009	196886879-198874600	192235076-193551650	within 3 Mb distance
chr4	5.0572	4p16.1-16.2	Giglio 2002	3792970-9461815		
chr7	0.9615	7p22.1	Feuk 2005	5832188-6899188		
chr7	0.0179	7q11.22	Feuk 2005	70058906-70076823		
chr7	2.2186	7q11.23	Osborne 2001	71956869-74995982		
chr8	4.6117	8p23.1	Giglio 2002	6913382-12332070	8804291-10982030	exten. search
chr9	23.5	9p12-q13	Starke 2002	37000000-71000000		
chr10	22.6	10p11.21-q21.1	Gilling 2006	37147500-59748500	37983987-57966260	each BP separately
chr15	5.9972	15q11.2-13.1	Gimelli 2003	20459937-27687533	26039213-27099713	right BP only
chr15	2	15q13.3	Antonacci 2009	28524207-30602466	27030510-27441289	within 1 MB distance
chr15	1.2	15q24	Antonacci 2009	72151413-73356183	72449440-73651390	
chr16	0.3052	16p11.2	Martin 2004	28256775-28695952	28234960-28804670	haplotype data only CEU
chr16	0.0011	16q24.1	Feuk 2005	83746238-83747302		
chr17	1.5	17q12	Antonacci 2009	31888441-33393152	34694635-35097385	within 1 Mb distance
chr17	0.9	17q21.31-21.32	Stefansson 2005	40930361- 1930361	41111654-42092850	

The table shows the details of 15 experimentally validated autosomic inversions, and their detection by a genome-wide scan on genotype data performed by inveRsion.

## Discussion

We proposed and implemented a method for detection of polymorphic inversions and classification of individuals into normal and inverted sub-populations for both phased haplotype and genotype data. We demonstrated the ability of our method to successfully detect inversions and classify individuals on simulations and showed how the performance of the method is impacted by the length and frequency of the inversion, SNP density, and the value of *t_B_*, the BIC threshold. Our findings demonstrate that genotype data can be used to detect inversions, although power for detection remains greater for haplotype data. Other approaches can be developed to perform inversion detection simultaneously with local haplotyping. In this work, we opted for a two-step strategy, which allows us to directly compare the impact of local haplotyping to inversion finding. As shown in Figure 2, the applicability of our approach is demonstrated by our performance tests showing, in particular, the small drop in accuracy we pay for treating genotype data, as compared to haplotype data.

Our formulation has several advantages compared to previously published methods to detect polymorphic inversions from nucleotide variation data [1,25]. Our work represents the first method capable of analyzing both genotype and haplotype data. Importantly, ours is the first LD based method to accurately classify individuals into normal and inverted subpopulations. By leveraging information from adjacent windows, our method is able to partition individuals into normal and inverted categories with high accuracy. Finally, our implementation is computationally efficient. Our use of a fixed size sliding-window to analyze chromosomes greatly improves upon the search efficiency of previous methods. In addition, a relevant computational improvement is achieved by the local phasing procedure of the genotype model. We tested the local phasing step of our algorithm on 60 CEUs (HapMap phase III) in a section of the chromosome 7 (0, 85 MB) with 42,379 SNPs. Using a 2.33 GHz intel Core 2 Duo, 2 GB RAM the analysis took only 40 minutes, which is ~ 13 times faster than phasing the whole segment with fastPhase. This shows that the load of a complete phasing of the data can be greatly reduced in the detection of genetic inversions. The overall efficiency of our method is an attractive feature for the analysis of already available data.

We illustrated the high accuracy (≥ 0.9) of our method over a wide range of inversion frequencies in principled-simulated data (Figure 4). We had difficulties successfully classifying individuals from invertFREGENE populations with inversion frequencies *>*80%. Note that in this scenario, high frequency inversions are affected by the age of the inversion and not by a simple change of reference genome. Within invertFREGENE, high frequency inversions tend to be older inversions, which may then present a higher within-population variability that can impact our predictions. However, the sudden drop of accuracy at high frequencies seems to indicate an additional critical behavior of the simulations at this range. Thus, in relation to the age of the inversion, the parameters used to run invertFREGENE may have not been optimized for this scenario.

In relation to real inversions, we show that the inveRsion package can be used to identify the inversion genotypes of subjects within the region 17q21. The high accuracies achieved for this inversion and the invertFREGENE simulations suggest that our method is most powerful when haplotype divergence is present. We also studied the inversion within 8p23. Despite of not detecting an inversion in this region in a genome-wide scan; with an extensive search, we were able to detect a signal (a third of that for the 17q21 region) and classify the subjects of the CEU population to a high degree. Consistently with Bosh et al. 2009, we saw that the most accurate predictions are made for the CEU population only. The 8p23 region has shown a number of complexities that might affect our inference to all HapMap groups. It has been shown that the region is unstable, showing mosiac rearrangements in its vicinity [24,29]. In addition, there is a lack of long distance LD, expected for two divergent haplotypes associated to each inverted status. Antonacci and colleagues suggested that this can be due to multiple inversion events. In addition, Deng et al. 2008 showed that the CEU population has an extended linkage disequilibrium in the region greater than that for the CBH+JPT and YRI populations. It is possible that our method particularly favors inversions with less variability within populations. Greater variability of populations within the inverted region could reduce our classification power because large groups of identical haoplotypes are harder to form. Further improvements of our method in this direction could improve performance.

An important practical consideration when using inveRsion is the threshold *BIC *value used to perform inversion genotyping. Larger values of *BIC *(Figure 2) do not always mean higher accuracy. As above, we recommend careful benchmarking of threshold *BIC *values for assessing subject classification. In the case of inferences in large populations and when experimental data is available on few subjects, a suitable threshold can be set for the classification that maximizes the inference on those subjects. We use this approach in our analysis of the 17q21 region, where we conducted a detailed search in a region known to have the inversion. When experimental data is not available, the stability of inversion allele for each subject could be studied for different *BIC *thresholds, drawing more confidence from those individuals that are consistently classified.

On a genome scale, initial scans can be conducted on several window sizes to identify regions of interest with positive *BIC *values where, as suggested by our simulations, we have high sensitivity. In our whole genome search for the CEU+YRI populations, we found recurrent inversions at different window sizes, which increases the confidence in the findings. This shows that using only three windows sizes, 0.4 - 0.7 - 1 Mb, can detect inversions from 0.4 Mb to ~ 2 Mb with enrichment for detecting inversions with length 0.7 - 1.4 Mb since more than one window have power to detect inversions in this range. The identification of recurrent inversions with different window sizes could also be used as an additional check to reduce false discoveries. In addition, these results suggest that scans with a few window sizes and as large a value of *L *as computationally feasible should be sufficient for a complete search for inversion signals in a given chromosome. Note that the window size is a probe for the detection of the inversion, thus it should be considered a function of the length of inversions to be detected. The accuracy of our method increases with higher SNP density. At the 500 k array density, our method can still achieve high classification accuracy. However, due to the high variability of the accuracy estimates, we recommend using imputed data in this case. To have a more accurate measure of the subject classification and length of the inversion, an extensive search for all possible lengths around the regions of interest can then be conducted.

Experimental information about the existence of inversions and their status in a sample of subjects is limited, constraining an exhaustive evaluation of the true positive and negative rates of our methodology with validated inversions. Inversions are difficult to characterize even with the newest sequencing data. In the study of structural variants in 1000 genomes, Durbin et al. 2010 [16] did not report inversions. Large inverted repeats at the breakpoints is a current obstacle for inversion detection using next generation sequencing data, in addition to the computational capacity to analyze hundreds of individuals. However, when inversion detection algorithms for sequence data are available, a necessary future evaluation of our methodology should be performed against this new technology.

## Conclusions

In this article, we have presented a methodology to detect inversion polymorphisms using nucleotide variation data from SNP micro-arrays. The method, based on LD differences across break points, is able to classify individuals into their inversion genotype status, allowing the gathering of population-based evidence of known inversions. In addition, using a computationally efficient scan across the genome, it provides evidence on new candidate regions that might be present in the population from which genotyped data is collected.

## Authors' contributions

AC devised the application of the inversion model to genotypes, work in the implementation of the software and co-wrote the manuscript, SSS formulated and implemented the inversion model for haplotypes, performed simulations, and co-wrote the manuscript, BJR work in the formulation of the inversion model and revised the manuscript, MC guided the analysis on real data, tested the model and revised the manuscript, JRG contributed to the implementation of the model, analysis strategies, revised the manuscript and coordinated the research. All authors read and approved the final manuscript.

## Supplementary Material

Additional file 1 **Additional Figures S1, S2, S3, S4, S5 and Tables S1, S2, S3, S4**. Click here for file
