# Supplementary material for: Identification of polymorphic inversions from genotypes
Source: BMC Bioinformatics. 2012 Feb 9;13:28. doi: 10.1186/1471-2105-13-28 (PMC3296650; doi:10.1186/1471-2105-13-28)
Supplement: Additional file 1 — Additional Figures S1, S2, S3, S4, S5 and Tables S1, S2, S3, S4. [file 1471-2105-13-28-S1.PDF]

Additional File 1

Identification of Polymorphic Inversions from Genotypes
Caceres et al. 2012

1 Supplementary Figure S1

Segmental sensitivity at different inversion lengths and population frequency of 60%.

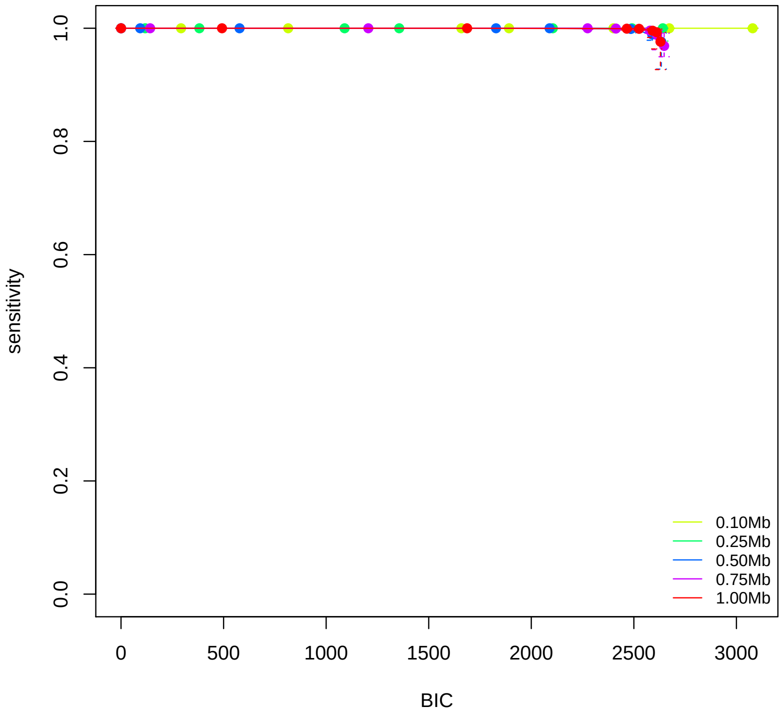

2 Supplementary Figure S2

Segmental FDR at different inversion lengths and population frequency of 60%.

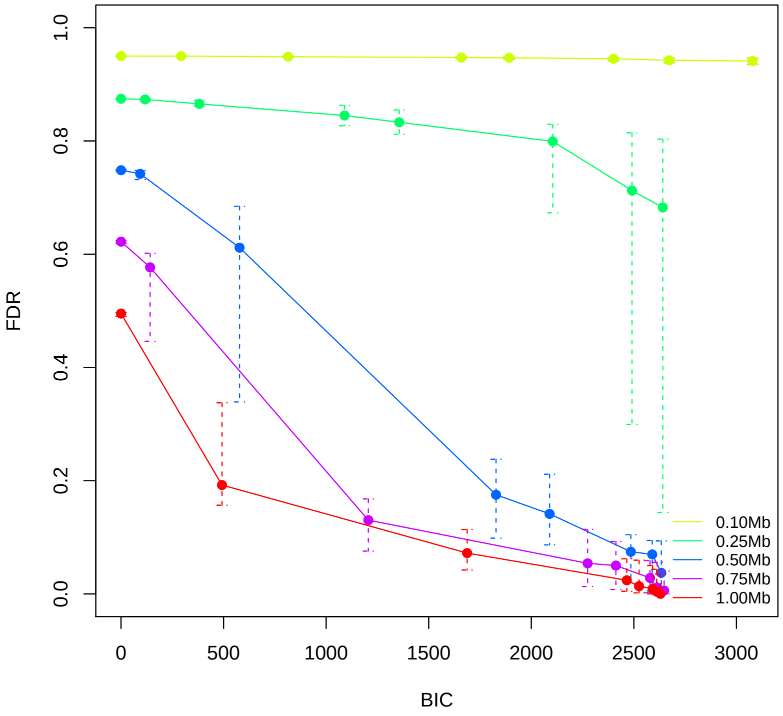

3 Supplementary Figure S3

Frequency of false regions of interest identified in a simulation with no inversions. 250 cases were run and scanned with window sizes of 0.2, 0.4 and 0.6Mb. We see that the method has less false positives regions of interest scanning high inversion lengths.

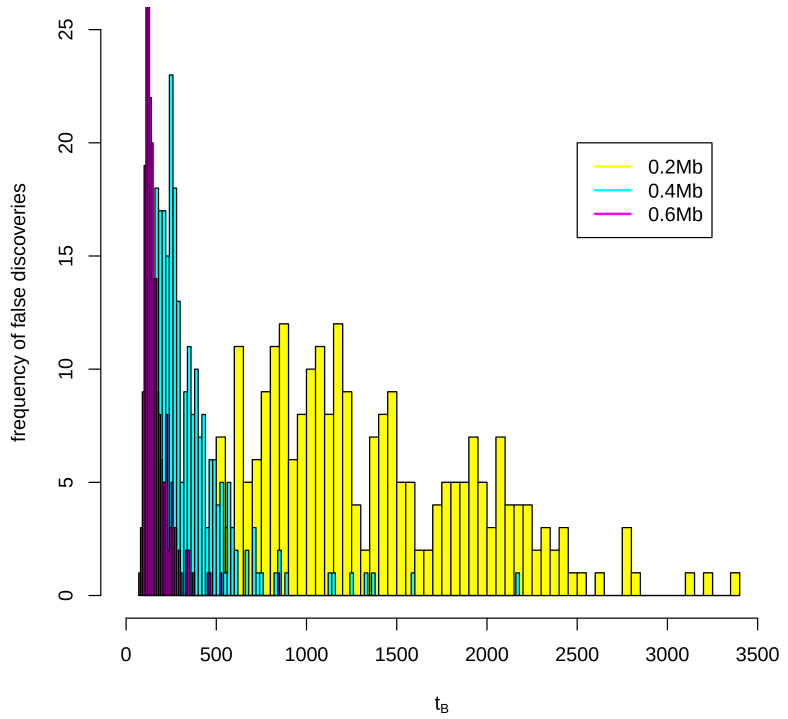

4 Supplementary Table S1

Table S1. Inverted sequences found in chromosome 16 of the CEU population genotypes reconstructed from the haplotypes. The inversions predictions on the raw genotypes (Table 1) were a subset of the predictions from the reconstructed genotypes and the phased haplotypes. As expected, the set of predictions from the reconstructed genotypes is more consistent with the predictions from the haplotypes than the raw genotypes. The difference in predictions for the two genotype sets reflects additional processing, such as data imputation, performed during the phasing process.

Genotypes*

window=0.4

28.24091	28.39264	28.73311	28.79316	112.12	0.59	28
34.07920	34.55067	34.48884	35.00029	256.76	0.28	64
45.70422	46.08432	46.21367	46.49861	177.31	0.78	18
68.51016	68.66370	68.93968	69.06441	165.09	0.42	106

window=0.7

33.74401	34.21972	34.46486	34.92703	134.54	0.44	15

5 Supplementary Figure S4

Inversion detected in the 17q21 after an extensive search between 39-43Mb. Overlaid at the the bottom, we show the SNP density. Gaps in the density coincide with known segmental duplications.

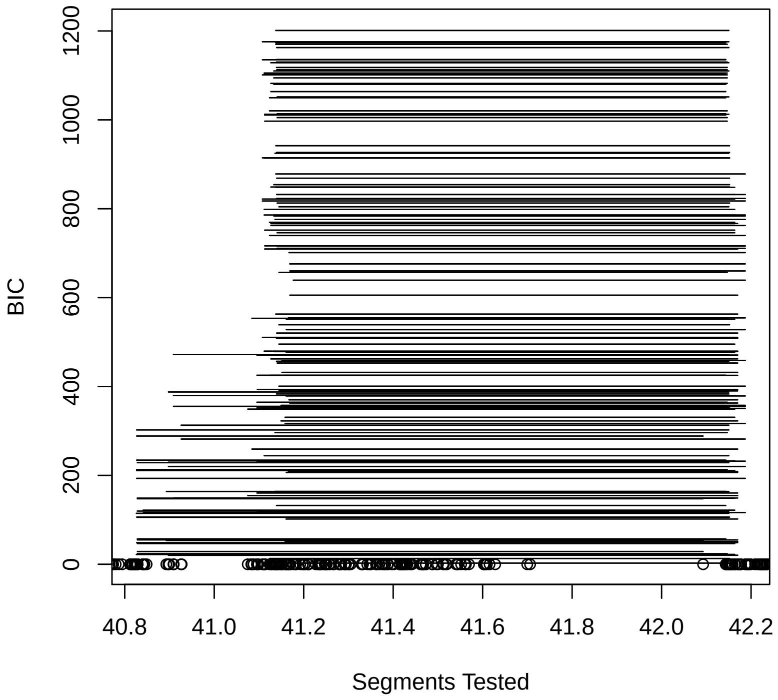

6 Supplementary Table S2

Inversion genotypes of 24 subjects as predicted by `inveRsion` compared to the reported values (Exp); see main text for references. (non-inverted homozygous: 0, inverted heterozygous: 1, inverted homozygous: 2) There is perfect concordance between `inveRsion` predictions and reported values.

	pop	ID	inveRsion	Exp
1	CEU	NA10847	1	1
2	CEU	NA11832	0	0
3	CEU	NA11840	0	0
4	CEU	NA1199	0	0
5	CEU	NA12156	1	1
6	CEU	NA12813	1	1
7	CEU	NA12878	0	0
8	YRI	NA18507	0	0
9	YRI	NA18517	0	0
10	CHB	NA18552	0	0
11	CHB	NA18555	0	0
12	CHB	NA18564	0	0
13	CHB	NA18573	0	0
14	YRI	NA18861	0	0
15	JPT	NA18942	0	0
16	JPT	NA18947	0	0
17	JPT	NA18956	0	0
18	JPT	NA18980	0	0
19	YRI	NA19102	0	0
20	YRI	NA19116	0	0
21	YRI	NA19129	0	0
22	YRI	NA19132	0	0
23	YRI	NA19172	0	0
24	YRI	NA19240	0	0

7 Supplementary Figure S5

Inversion detected in the 8p23 after an extensive search between 6-13Mb. Overlaid at the the bottom, we show the SNP density. Gaps in the density coincide with known segmental duplications.

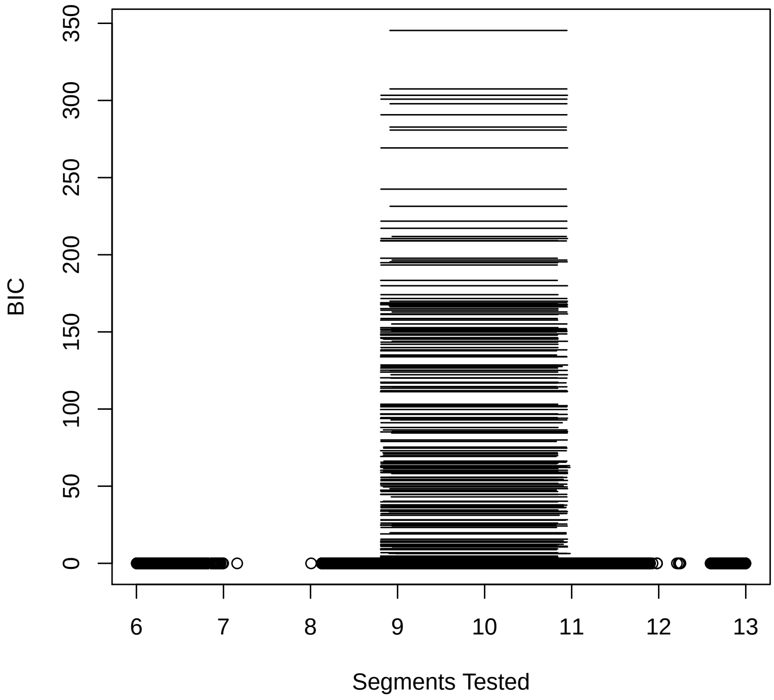

8 Supplementary Table S3

Inversion genotypes of 41 subjects as predicted by `inveRsion` compared to the reported values (Exp); see main text for references. (non-inverted homozygous: 0, inverted heterozygous: 1, inverted homozygous: 2)

	pop	ID	inveRsion	Exp
1	CEU	NA06985	2	2
2	CEU	NA06993	1	1
3	CEU	NA06994	2	2
4	CEU	NA07055	2	2
5	CEU	NA10847	1	1
6	CEU	NA11831	1	0
7	CEU	NA11832	0	0
8	CEU	NA11839	2	2
9	CEU	NA11840	2	1
10	CEU	NA11992	2	2
11	CEU	NA11993	1	1
12	CEU	NA11994	1	1
13	CEU	NA12057	2	2
14	CEU	NA12155	0	0
15	CEU	NA12156	1	1
16	CEU	NA12249	0	0
17	CEU	NA12264	2	2
18	CEU	NA12813	1	1
19	CEU	NA12815	0	0
20	CEU	NA12878	2	2
21	YRI	NA18507	2	1
22	YRI	NA18517	2	1
23	CHB	NA18529	0	1
24	CHB	NA18552	0	0
25	CHB	NA18555	0	1
26	CHB	NA18564	0	1
27	CHB	NA18571	0	1
28	CHB	NA18573	0	0
29	YRI	NA18852	2	2
30	YRI	NA18853	2	2
31	YRI	NA18861	2	1
32	JPT	NA18942	0	0
33	JPT	NA18947	0	0
34	JPT	NA18956	0	0

35	JPT	NA18980	0	0
36	YRI	NA19102	2	1
37	YRI	NA19116	2	1
38	YRI	NA19129	2	1
39	YRI	NA19132	2	2
40	YRI	NA19172	2	1
41	YRI	NA19240	2	2

9 Supplementary Table S4

We report 263 predicted inversion regions from a genome-wide scan of the CEU and YRI populations of HapMap III. We used three window sizes (1Mb, 0.7Mb and 0.4Mb) and reported regions detected from overlapping windows with $t_B > 0$. Here we report the outer limits of of the left and right break points of the inversions found. We aggregate the inversions according to their recurrent detection by each window size, and report the window size with the highest number of significant windows (nr). The right most columns (`val`, `val_1Mb`) indicate whether they have been reported in previous experimental studies (K: Kidd 2008 and L: Levy 2007). We find a total of 52 inversions, 20% our predicted regions, that overlap either Kidd's or Levy's inversions (`val`). A higher proportion, 89 inversions (33 %), are at least 1MB distance from reported ones (`val_1Mb`).

CHROMOSOME 1

	LBP_win1	RBP_win1	LBP_win0.7	RBP_win0.7	LBP_win0.4	RBP_win0.4	nr	val	val_1Mb
1	49102291	50343252	49102291	50361252	49102291	50361252	1055 (0.4)	--	--
2	35299265	36778343	NA	NA	NA	NA	33 (1)	--	--
3	171764640	173247134	171216470	172864171	172034066	172952384	25 (1)	--	K-
4	NA	NA	210834463	211910671	NA	NA	106 (0.7)	--	--
5	NA	NA	116245617	117194010	116576599	117146711	76 (0.4)	--	--
6	NA	NA	194805535	196001522	195596158	196078189	22 (0.7)	-L	-L
7	NA	NA	NA	NA	45707643	46394327	965 (0.4)	--	--
8	NA	NA	NA	NA	50625356	51481371	441 (0.4)	--	--
9	NA	NA	NA	NA	35298689	36489407	382 (0.4)	--	--
10	NA	NA	NA	NA	72974957	73673535	314 (0.4)	--	--
11	NA	NA	NA	NA	92256403	93136655	270 (0.4)	--	--
12	NA	NA	NA	NA	52187515	52876476	100 (0.4)	--	--
13	NA	NA	NA	NA	45040943	45536129	59 (0.4)	--	--
14	NA	NA	NA	NA	152094704	152796045	43 (0.4)	--	K-
15	NA	NA	NA	NA	77804582	78346717	29 (0.4)	--	--
16	NA	NA	NA	NA	20927313	21388493	22 (0.4)	--	--
17	NA	NA	NA	NA	207905997	208357932	18 (0.4)	--	--
18	NA	NA	NA	NA	8359389	8794277	17 (0.4)	--	--
19	NA	NA	NA	NA	153485070	154177454	15 (0.4)	--	--
20	NA	NA	NA	NA	890593	1487064	9 (0.4)	K-	KL
21	NA	NA	NA	NA	28690184	29244086	9 (0.4)	--	--
22	NA	NA	NA	NA	102901433	103364497	6 (0.4)	--	--
23	NA	NA	NA	NA	32330073	32795137	2 (0.4)	--	--
24	NA	NA	NA	NA	148669237	149127095	1 (0.4)	--	K-
25	NA	NA	NA	NA	184510205	184912550	1 (0.4)	--	-L

CHROMOSOME 2

	LBP_win1	RBP_win1	LBP_win0.7	RBP_win0.7	LBP_win0.4	RBP_win0.4	nr	val	val_1Mb
1	135246172	136616329	135407661	136319746	135011650	136616329	70 (1)	--	K-
2	95398090	96827256	94944592	96440369	94992765	97591869	55 (0.7)	K-	K-
3	NA	NA	96920270	97828234	94992765	97591869	125 (0.7)	K-	K-
4	NA	NA	218834980	219703391	219131849	219553299	3 (0.7)	--	--

5	NA	NA	NA	NA	186597505	187136355	230 (0.4)	--	--
6	NA	NA	NA	NA	31610528	32342662	176 (0.4)	--	--
7	NA	NA	NA	NA	27439659	27996521	12 (0.4)	--	--
8	NA	NA	NA	NA	110258184	110856557	12 (0.4)	K-	K-
9	NA	NA	NA	NA	61157775	61690277	8 (0.4)	K-	K-
10	NA	NA	NA	NA	189592832	190030441	5 (0.4)	--	--
11	NA	NA	NA	NA	161915756	162375413	3 (0.4)	--	--
12	NA	NA	NA	NA	186025907	186504660	3 (0.4)	--	--

CHROMOSOME 3

	LBP_win1	RBP_win1	LBP_win0.7	RBP_win0.7	LBP_win0.4	RBP_win0.4	nr	val	val_1Mb
1	120164552	121959848	NA	NA	NA	NA	120 (1)	--	--
2	110572809	113538483	111753973	112532936	111802392	112664903	1040 (0.4)	--	--
3	46549422	52303573	46999499	51568867	46999499	51568867	97 (1)	KL	KL
4	96362581	98652626	95708476	98735243	95824973	97260789	88 (1)	--	--
5	96362581	98652626	95708476	98735243	97829661	98774726	88 (1)	--	--
6	168647046	170154427	NA	NA	NA	NA	66 (1)	--	K-
7	5034576	7185728	NA	NA	NA	NA	38 (1)	--	--
8	80546372	82079507	80471418	81181690	80362995	81282396	531 (0.4)	--	--
9	130749747	132060426	129642049	133536739	129988519	131434785	59 (0.7)	--	--
10	130749747	132060426	129642049	133536739	131974495	132605421	59 (0.7)	--	--
11	130749747	132060426	129642049	133536739	133234465	133705647	59 (0.7)	--	--
12	26214856	27267561	25553896	26677984	25730717	27545290	427 (0.4)	--	--
13	192235076	193551650	NA	NA	NA	NA	5 (1)	--	--
14	138833008	140324166	NA	NA	NA	NA	4 (1)	--	--
15	83237104	84638895	84638895	86441265	84077570	84886996	38 (0.4)	--	--
16	83237104	84638895	84638895	86441265	85049557	86517899	18 (0.7)	--	--
17	183268442	184827217	NA	NA	NA	NA	1 (1)	--	--
18	NA	NA	115541103	116640412	116013369	116596467	19 (0.7)	--	--
19	NA	NA	42630050	43668282	NA	NA	9 (0.7)	--	--
20	NA	NA	137164904	138583953	137164904	138033238	9 (0.7)	--	--
21	NA	NA	143553185	144511780	NA	NA	6 (0.7)	--	--
22	NA	NA	89499861	90235192	89499861	90510976	35 (0.4)	--	--
23	NA	NA	NA	NA	17221396	17857672	894 (0.4)	--	--
24	NA	NA	NA	NA	44352963	45051827	710 (0.4)	-L	-L
25	NA	NA	NA	NA	52303573	53203382	321 (0.4)	--	--
26	NA	NA	NA	NA	57055753	57920283	320 (0.4)	-L	-L
27	NA	NA	NA	NA	83027115	83781723	178 (0.4)	--	--
28	NA	NA	NA	NA	81872980	82471376	150 (0.4)	--	--
29	NA	NA	NA	NA	95071254	95702121	104 (0.4)	--	--
30	NA	NA	NA	NA	45568973	46838221	79 (0.4)	--	-L
31	NA	NA	NA	NA	159376137	159977392	44 (0.4)	--	--
32	NA	NA	NA	NA	15458834	15965882	41 (0.4)	--	--
33	NA	NA	NA	NA	120846256	121565472	37 (0.4)	--	--
34	NA	NA	NA	NA	11988512	12418657	31 (0.4)	K-	K-
35	NA	NA	NA	NA	182046241	182592762	23 (0.4)	--	--
36	NA	NA	NA	NA	36951930	37412308	15 (0.4)	--	--
37	NA	NA	NA	NA	33412548	33828751	14 (0.4)	--	-L
38	NA	NA	NA	NA	183784167	184574024	11 (0.4)	--	--
39	NA	NA	NA	NA	156628046	157384963	9 (0.4)	--	--
40	NA	NA	NA	NA	163785970	164457794	8 (0.4)	-L	-L
41	NA	NA	NA	NA	162206058	162737491	5 (0.4)	--	--
42	NA	NA	NA	NA	165186095	165710488	5 (0.4)	--	--
43	NA	NA	NA	NA	145302196	145781140	3 (0.4)	--	--
44	NA	NA	NA	NA	167471088	167897919	1 (0.4)	--	--

CHROMOSOME 4

	LBP_win1	RBP_win1	LBP_win0.7	RBP_win0.7	LBP_win0.4	RBP_win0.4	nr	val	val_1Mb
1	172268772	173592679	NA	NA	NA	NA	51 (1)	--	K-
2	32774763	33894054	32774763	34012465	33423803	34190319	540 (0.4)	--	--
3	NA	NA	NA	NA	151611156	152389749	1472 (0.4)	--	--
4	NA	NA	NA	NA	48048814	48786539	159 (0.4)	--	K-
5	NA	NA	NA	NA	18759836	19326552	114 (0.4)	--	--
6	NA	NA	NA	NA	52805422	53419501	49 (0.4)	--	--
7	NA	NA	NA	NA	128316065	129244493	26 (0.4)	--	-L
8	NA	NA	NA	NA	103931669	104582199	23 (0.4)	--	--
9	NA	NA	NA	NA	52378364	52802795	19 (0.4)	--	--
10	NA	NA	NA	NA	85785833	86287914	14 (0.4)	--	--
11	NA	NA	NA	NA	98378031	99169984	11 (0.4)	--	--
12	NA	NA	NA	NA	152488678	152946749	3 (0.4)	--	--

CHROMOSOME 5

	LBP_win1	RBP_win1	LBP_win0.7	RBP_win0.7	LBP_win0.4	RBP_win0.4	nr	val	val_1Mb
1	130204101	131695635	130204101	131695635	NA	NA	41 (0.7)	--	--
2	45312869	46419092	45312869	46419092	NA	NA	7 (1)	--	--
3	NA	NA	36281189	37685399	36775124	37789866	339 (0.4)	--	--
4	NA	NA	60100142	60863726	60036227	60747389	435 (0.4)	--	--
5	NA	NA	NA	NA	41639742	42478472	857 (0.4)	--	--
6	NA	NA	NA	NA	87332273	87986046	330 (0.4)	--	--
7	NA	NA	NA	NA	12148032	12672289	186 (0.4)	--	--
8	NA	NA	NA	NA	43406123	43898451	31 (0.4)	--	--
9	NA	NA	NA	NA	49635303	50089301	19 (0.4)	--	--
10	NA	NA	NA	NA	61472068	61976799	7 (0.4)	--	--

CHROMOSOME 6

	LBP_win1	RBP_win1	LBP_win0.7	RBP_win0.7	LBP_win0.4	RBP_win0.4	nr	val	val_1Mb
1	26501000	29414635	26343945	29533987	26484140	29591947	648 (1)	K-	K-
2	NA	NA	29653187	31114071	29724586	31004378	414 (0.7)	--	-L
3	NA	NA	62848148	64081983	62819273	63814587	164 (0.4)	--	K-
4	NA	NA	NA	NA	34665224	35470826	168 (0.4)	--	K-
5	NA	NA	NA	NA	62042822	62735203	134 (0.4)	K-	K-
6	NA	NA	NA	NA	31179037	31650287	104 (0.4)	--	-L
7	NA	NA	NA	NA	31723146	32808826	94 (0.4)	--	-L
8	NA	NA	NA	NA	146171040	146901427	30 (0.4)	--	--
9	NA	NA	NA	NA	78380388	78982202	9 (0.4)	--	--
10	NA	NA	NA	NA	44941576	45400416	1 (0.4)	--	--
11	NA	NA	NA	NA	126702847	127121034	1 (0.4)	--	K-

CHROMOSOME 7

	LBP_win1	RBP_win1	LBP_win0.7	RBP_win0.7	LBP_win0.4	RBP_win0.4	nr	val	val_1Mb
1	64087991	65866167	64407931	65868295	NA	NA	265 (0.7)	K-	K-
2	NA	NA	97645818	99475178	98618456	99935831	201 (0.7)	--	--
3	NA	NA	61078136	62120698	NA	NA	16 (0.7)	K-	K-
4	NA	NA	118531269	119254276	118526094	119493023	71 (0.4)	K-	K-
5	NA	NA	NA	NA	62336389	62887215	14 (0.4)	K-	K-
6	NA	NA	NA	NA	56237968	56668472	5 (0.4)	--	K-

7	NA	NA	NA	NA	85446017	85892065	1 (0.4)	--	--

CHROMOSOME 8

	LBP_win1	RBP_win1	LBP_win0.7	RBP_win0.7	LBP_win0.4	RBP_win0.4	nr	val	val_1Mb
1	47056005	50943107	47043376	49438908	47043376	49369296	745 (0.7)	KL	KL
2	47056005	50943107	50816259	51552222	50342155	51479247	153 (1)	KL	KL
3	42344173	43870532	42772016	43881838	42772016	43881838	65 (1)	--	--
4	NA	NA	67080630	68551613	NA	NA	26 (0.7)	--	--
5	NA	NA	78422402	79263139	NA	NA	13 (0.7)	--	--
6	NA	NA	112558898	113387234	112811786	113467267	32 (0.4)	--	--
7	NA	NA	NA	NA	52427538	53251464	278 (0.4)	--	--
8	NA	NA	NA	NA	114100169	114641713	13 (0.4)	--	--
9	NA	NA	NA	NA	99920891	100484483	2 (0.4)	--	--

CHROMOSOME 9

	LBP_win1	RBP_win1	LBP_win0.7	RBP_win0.7	LBP_win0.4	RBP_win0.4	nr	val	val_1Mb
1	123918828	125965852	123997346	127564855	125930668	127756213	810 (1)	K-	K-
2	97614274	99358852	97919978	99127041	98386391	99015827	84 (0.4)	--	--
3	106724074	107952732	NA	NA	NA	NA	17 (1)	--	--
4	14725694	16000931	15744393	16498227	15405461	16351580	511 (0.4)	--	--
5	NA	NA	122889993	123775902	NA	NA	23 (0.7)	--	K-
6	NA	NA	87108051	88332725	NA	NA	17 (0.7)	--	--
7	NA	NA	129886930	130787532	129753437	130639932	45 (0.4)	--	--
8	NA	NA	96101365	97211906	96515217	97104587	15 (0.4)	--	--
9	NA	NA	13481120	14593494	NA	NA	1 (0.7)	--	--
10	NA	NA	NA	NA	11140966	11997658	754 (0.4)	--	--
11	NA	NA	NA	NA	93984599	94730858	388 (0.4)	--	--
12	NA	NA	NA	NA	30266852	30889622	257 (0.4)	--	--
13	NA	NA	NA	NA	128635716	129416108	17 (0.4)	--	--
14	NA	NA	NA	NA	33752135	34214285	15 (0.4)	--	--
15	NA	NA	NA	NA	101851624	102383222	9 (0.4)	--	--
16	NA	NA	NA	NA	89669105	90279532	6 (0.4)	--	--
17	NA	NA	NA	NA	75184352	75714365	2 (0.4)	--	-L

CHROMOSOME 10

	LBP_win1	RBP_win1	LBP_win0.7	RBP_win0.7	LBP_win0.4	RBP_win0.4	nr	val	val_1Mb
1	73493746	75271694	73519478	74867057	73493746	75003198	473 (0.4)	K-	K-
2	30308526	31387790	31383794	32408538	NA	NA	10 (1)	--	--
3	37983987	39090896	37817283	39090896	NA	NA	61 (0.7)	--	K-
4	NA	NA	102696588	103738323	103357246	103998270	36 (0.4)	--	--
5	NA	NA	NA	NA	68606231	69466340	297 (0.4)	--	--
6	NA	NA	NA	NA	21822848	22400452	107 (0.4)	--	--
7	NA	NA	NA	NA	41753546	42504435	66 (0.4)	--	--
8	NA	NA	NA	NA	57447489	57966260	63 (0.4)	--	-L
9	NA	NA	NA	NA	32840772	33321628	32 (0.4)	--	--
10	NA	NA	NA	NA	104651474	105196864	13 (0.4)	--	--
11	NA	NA	NA	NA	116702319	117440711	1 (0.4)	K-	K-

CHROMOSOME 11

	LBP_win1	RBP_win1	LBP_win0.7	RBP_win0.7	LBP_win0.4	RBP_win0.4	nr	val	val_1Mb

1	54592199	56081152	54537133	56166179	54596847	56299504	1631 (0.7)	K-	K-
2	47158500	51078178	NA	NA	NA	NA	6 (1)	KL	KL
3	NA	NA	NA	NA	14288203	14873873	255 (0.4)	--	--
4	NA	NA	NA	NA	37939871	38589346	169 (0.4)	--	--
5	NA	NA	NA	NA	27902922	28521750	76 (0.4)	--	--
6	NA	NA	NA	NA	65872358	67154251	34 (0.4)	K-	K-
7	NA	NA	NA	NA	9779172	10287887	18 (0.4)	--	K-
8	NA	NA	NA	NA	31021146	31659092	11 (0.4)	--	--
9	NA	NA	NA	NA	57156087	57690523	5 (0.4)	--	--
10	NA	NA	NA	NA	57748644	58184159	4 (0.4)	--	--
11	NA	NA	NA	NA	110922989	111682348	1 (0.4)	--	--

CHROMOSOME 12

	LBP_win1	RBP_win1	LBP_win0.7	RBP_win0.7	LBP_win0.4	RBP_win0.4	nr	val	val_1Mb
1	36144018	38417007	36144018	38269149	36144018	38417007	1761 (0.7)	--	--
2	110268878	111894364	110062487	111509176	109947738	111717784	1395 (1)	--	K-
3	119405544	122312914	119324905	122943474	119479715	120366231	1690 (0.7)	K-	K-
4	119405544	122312914	119324905	122943474	120634766	122466089	1690 (0.7)	K-	K-
5	53622266	56140578	54181381	55880161	54080531	55193713	492 (0.4)	--	--
6	78494470	79850724	78022922	79587089	78022922	79006209	298 (0.4)	KL	KL
7	20099882	22138851	NA	NA	NA	NA	182 (1)	--	--
8	32587277	36144018	36144018	38269149	36144018	38417007	1761 (0.7)	--	--
9	32587277	36144018	32995423	34727104	33049704	34711193	1044 (0.7)	--	--
10	50678467	52213821	50861047	52825272	51724159	52840967	514 (0.7)	K-	K-
11	47203274	48887422	48364391	49734791	47764925	49416474	334 (0.7)	--	--
12	63616027	65212726	NA	NA	NA	NA	13 (1)	--	--
13	43101972	44329691	NA	NA	NA	NA	2 (1)	--	--
14	106151673	107688511	NA	NA	NA	NA	2 (1)	K-	K-
15	NA	NA	29658401	31029585	NA	NA	146 (0.7)	--	--
16	NA	NA	6338564	7492246	NA	NA	18 (0.7)	--	K-
17	NA	NA	42114933	43100050	42111682	42981260	53 (0.4)	--	--
18	NA	NA	86849619	87855513	86982827	87874101	609 (0.4)	--	--
19	NA	NA	85152399	85899115	84889921	85902122	92 (0.4)	KL	KL
20	NA	NA	NA	NA	28135765	28635357	57 (0.4)	--	--
21	NA	NA	NA	NA	82895046	83490305	14 (0.4)	--	--
22	NA	NA	NA	NA	45045403	45610863	11 (0.4)	--	--
23	NA	NA	NA	NA	72372101	73027225	11 (0.4)	--	--
24	NA	NA	NA	NA	58515124	59405533	7 (0.4)	--	--
25	NA	NA	NA	NA	40642059	41048753	2 (0.4)	--	--

CHROMOSOME 13

	LBP_win1	RBP_win1	LBP_win0.7	RBP_win0.7	LBP_win0.4	RBP_win0.4	nr	val	val_1Mb
1	NA	NA	NA	NA	54520028	55243360	322 (0.4)	--	--
2	NA	NA	NA	NA	51422561	52124119	179 (0.4)	K-	K-
3	NA	NA	NA	NA	56460128	57132165	140 (0.4)	--	--
4	NA	NA	NA	NA	55598251	56355405	106 (0.4)	--	--
5	NA	NA	NA	NA	86115979	86534975	9 (0.4)	--	--
6	NA	NA	NA	NA	95104147	95706723	9 (0.4)	K-	K-
7	NA	NA	NA	NA	88114112	88562964	4 (0.4)	--	--

CHROMOSOME 14

	LBP_win1	RBP_win1	LBP_win0.7	RBP_win0.7	LBP_win0.4	RBP_win0.4	nr	val	val_1Mb
1	65689881	66956534	65588815	66947878	65689881	66954923	5541 (0.7)	--	K-
2	NA	NA	NA	NA	36570910	37173757	422 (0.4)	--	--
3	NA	NA	NA	NA	38529823	39045678	25 (0.4)	--	--
4	NA	NA	NA	NA	59646390	60332193	13 (0.4)	K-	K-
5	NA	NA	NA	NA	54441329	54941205	3 (0.4)	--	--

CHROMOSOME 15

	LBP_win1	RBP_win1	LBP_win0.7	RBP_win0.7	LBP_win0.4	RBP_win0.4	nr	val	val_1Mb
1	40397858	44373066	39930204	43440659	40360359	42014562	1943 (1)	K-	K-
2	40397858	44373066	39930204	43440659	42746424	43682118	1943 (1)	K-	K-
3	40397858	44373066	39930204	43440659	42267001	42731049	1943 (1)	K-	K-
4	26039213	27099713	NA	NA	NA	NA	186 (1)	K-	K-
5	NA	NA	74376550	75256074	74281930	75094965	1643 (0.4)	--	K-
6	NA	NA	74376550	75256074	75155737	75593852	178 (0.7)	--	--
7	NA	NA	69990571	71145004	69899972	70945211	368 (0.4)	K-	K-
8	NA	NA	NA	NA	62236892	62894812	229 (0.4)	--	--
9	NA	NA	NA	NA	54759509	55368884	180 (0.4)	--	K-
10	NA	NA	NA	NA	47135746	47762188	142 (0.4)	--	--
11	NA	NA	NA	NA	82496114	82987641	80 (0.4)	K-	K-
12	NA	NA	NA	NA	72449440	73651390	40 (0.4)	K-	K-
13	NA	NA	NA	NA	26032853	26740359	31 (0.4)	K-	K-
14	NA	NA	NA	NA	27030510	27441289	2 (0.4)	--	K-

CHROMOSOME 16

	LBP_win1	RBP_win1	LBP_win0.7	RBP_win0.7	LBP_win0.4	RBP_win0.4	nr	val	val_1Mb
1	65379967	66826337	65519912	66958878	65385348	66935349	2159 (0.7)	--	--
2	45652351	46730882	45635029	46702190	45160320	46512332	258 (0.7)	--	--
3	NA	NA	34079200	34802728	34079200	34935609	14 (0.4)	K-	K-
4	NA	NA	NA	NA	68503033	69080511	161 (0.4)	K-	K-
5	NA	NA	NA	NA	14540007	15047712	64 (0.4)	K-	K-
6	NA	NA	NA	NA	70751826	71454195	38 (0.4)	K-	K-

CHROMOSOME 17

	LBP_win1	RBP_win1	LBP_win0.7	RBP_win0.7	LBP_win0.4	RBP_win0.4	nr	val	val_1Mb
1	55074583	56516428	55074583	56706403	55074583	56610013	702 (1)	KL	KL
2	24089338	26354670	25059140	26354670	24968215	25917773	391 (1)	K-	K-
3	24089338	26354670	25059140	26354670	26321721	26756090	391 (1)	K-	K-
4	41111654	42187100	41111654	42191820	41112752	42092850	374 (0.7)	K-	KL
5	NA	NA	70039686	71514525	NA	NA	361 (0.7)	--	--
6	NA	NA	58107636	59760703	59613108	60378951	72 (0.7)	--	-L
7	NA	NA	58107636	59760703	58653742	59269058	72 (0.7)	--	-L
8	NA	NA	53786529	54695977	53786529	54707682	2612 (0.4)	--	KL
9	NA	NA	NA	NA	21891848	22525990	223 (0.4)	--	--
10	NA	NA	NA	NA	19772411	20231611	21 (0.4)	--	K-
11	NA	NA	NA	NA	38359611	38826209	11 (0.4)	--	--
12	NA	NA	NA	NA	18841513	19293970	9 (0.4)	K-	K-
13	NA	NA	NA	NA	43407792	43880182	4 (0.4)	--	--
14	NA	NA	NA	NA	34694635	35097385	1 (0.4)	--	--

CHROMOSOME 18

	LBP_win1	RBP_win1	LBP_win0.7	RBP_win0.7	LBP_win0.4	RBP_win0.4	nr	val	val_1Mb
1	NA	NA	16881028	18524142	16803435	17797270	1134 (0.4)	--	--
2	NA	NA	NA	NA	49566525	50049351	19 (0.4)	--	--
3	NA	NA	NA	NA	32605527	33074986	2 (0.4)	--	K-

[1] " CHROMOSOME 19"

	LBP_win1	RBP_win1	LBP_win0.7	RBP_win0.7	LBP_win0.4	RBP_win0.4	nr	val	val_1Mb
1	NA	NA	46426400	47797213	46693050	47698080	283 (0.4)	--	--
2	NA	NA	NA	NA	23730401	24313463	9 (0.4)	--	--
3	NA	NA	NA	NA	41632600	43029577	8 (0.4)	--	KL
4	NA	NA	NA	NA	32640702	33123797	1 (0.4)	--	--

CHROMOSOME 20

	LBP_win1	RBP_win1	LBP_win0.7	RBP_win0.7	LBP_win0.4	RBP_win0.4	nr	val	val_1Mb
1	29617898	33268937	NA	NA	NA	NA	544 (1)	--	--
2	NA	NA	25198577	26187964	25131908	26237925	1939 (0.4)	K-	K-
3	NA	NA	NA	NA	32070396	33063262	144 (0.4)	--	--
4	NA	NA	NA	NA	33523089	34019419	14 (0.4)	--	K-
5	NA	NA	NA	NA	31342234	31792629	1 (0.4)	--	--

CHROMOSOME 21

	LBP_win1	RBP_win1	LBP_win0.7	RBP_win0.7	LBP_win0.4	RBP_win0.4	nr	val	val_1Mb
1	17042335	18945713	NA	NA	NA	NA	4 (1)	--	--
2	NA	NA	28997768	29849643	28997768	29701814	128 (0.7)	--	--
3	NA	NA	NA	NA	17485166	17893230	1 (0.4)	--	--

CHROMOSOME 22

	LBP_win1	RBP_win1	LBP_win0.7	RBP_win0.7	LBP_win0.4	RBP_win0.4	nr	val	val_1Mb
1	39162321	41331072	NA	NA	NA	NA	265 (1)	--	--
2	37630882	38745971	NA	NA	NA	NA	20 (1)	--	--
3	NA	NA	26623980	27655923	26623980	27744001	576 (0.4)	--	K-
4	NA	NA	29982338	30779042	29875391	30748552	447 (0.4)	--	--
5	NA	NA	NA	NA	28265773	28931243	619 (0.4)	--	--
6	NA	NA	NA	NA	39899555	40899943	450 (0.4)	--	--
7	NA	NA	NA	NA	38873554	39655014	213 (0.4)	--	--
8	NA	NA	NA	NA	34158300	34834625	16 (0.4)	--	--
